# Selecting patients for magnetic resonance imaging cognitive versus ultrasound fusion biopsy of the prostate: A within‐patient comparison

**DOI:** 10.1002/bco2.172

**Published:** 2022-06-05

**Authors:** Mitch Hayes, Solange Bassale, Nicholas H. Chakiryan, Luc Boileau, Jacob Grassauer, Matthew Wagner, Bryan Foster, Fergus Coakley, Sudhir Isharwal, Christopher L. Amling, Jen‐Jane Liu

**Affiliations:** ^1^ Department of Urology Oregon Health & Science University Portland Oregon USA; ^2^ Knight Cancer Institute, Biostatistics Shared Resources Oregon Health and Science University Portland Oregon USA; ^3^ Department of Urology Kaiser Permanente Portland Oregon USA; ^4^ Department of Radiology Oregon Health and Science University Portland Oregon USA

**Keywords:** biopsy, diagnosis, magnetic resonance imaging, prostate cancer

## Abstract

**Objectives:**

To compare overall agreement between magnetic resonance imaging (MRI)–ultrasound (US) fusion biopsy (FB) and MRI cognitive fusion biopsy (CB) of the prostate and determine which factors affect agreement for prostate cancer (PCa) who underwent both modalities in a prospective within‐patient protocol.

**Patients and Methods:**

From August 2017 to January 2021, patients with at least one Prostate Imaging Reporting & Data System (PI‐RADS) 3 or higher lesion on multiparametric MRI underwent transrectal FB and CB in a prospective within‐patient protocol. CB was performed for each region of interest (ROI), followed by FB, followed by standard 12 core biopsy. Patients who were not on active surveillance were analysed. The primary endpoint was agreement for any PCa detection. McNemar's test and kappa statistic were used to analyse agreement. Chi‐square test, Fisher's exact test and Wilcoxon rank sum test were used to analyse disagreement across clinical and MRI spatial variables. A multivariable generalized mixed‐effect model was used to compare the interaction between select variables and fusion modality. Statistics were performed using SAS and R.

**Results:**

Ninety patients and 98 lesions were included in the analysis. There was moderate agreement between FB and CB (*k* = 0.715). McNemar's test was insignificant (*p* = 0.285). Anterior location was the only variable associated with a significant variation in agreement, which was 70% for anterior lesions versus 89.7% for non‐anterior lesions (*p* = 0.035). Discordance did not vary significantly across other variables. In a mixed‐effect model, the interaction between anterior location and use of FB was insignificant (*p* = 0.411).

**Conclusion:**

In a within‐patient protocol of patients not on active surveillance, FB and CB performed similarly for PCa detection and with moderate agreement. Anterior location was associated with significantly higher disagreement, whereas other patient and lesion characteristics were not. Additional studies are needed to determine optimal biopsy technique for sampling anterior ROI.

## 1 INTRODUCTION

Multiparametric prostate magnetic resonance imaging (MRI) (mpMRI) increases detection of high‐grade prostate cancer (PCa) and decreases detection of low‐grade cancers compared with standard template prostate biopsy.^1^, ^2^, ^3^, ^4^ To accurately target regions of interest (ROIs), fusion biopsy (FB) requires precise contouring by radiologists, and software‐guided co‐registration of the ROI and prostate contours onto transrectal ultrasound (TRUS) images. FB also requires capital investment to acquire the specialized technology necessary to overlay the images. Cognitive fusion biopsy (CB) allows targeting of MRI‐visible ROIs without FB technology. CB relies on the urologist's ability to accurately target the ROI without direct illustrated guidance from a co‐registered target. Prior studies comparing CB and FB have had mixed results. Some have shown similar detection rates for high‐grade PCa between CB and FB, whereas others have shown an advantage with FB.^5^, ^6^, ^7^, ^8^, ^9^, ^10^ Multiple systematic reviews and a meta‐analysis have not shown a difference in high‐grade cancer detection between CB and FB.^11^, ^12^

Thus, for diagnostic purposes, it is not clear what advantage software registration confers over simply knowing where an MRI suspicious ROI is located within the prostate. In patients with an elevated prostate‐specific antigen (PSA) and positive MRI, urologists who do not have access to FB technology must decide between proceeding with CB or referral to a centre with FB equipment. We are interested in determining if there are patient selection criteria that can optimally identify which technique to use. The primary objective of this study was to compare PCa detection rate between CB and FB and examine the effect of spatial parameters on performance of the two modalities side by side in patients who underwent both CB and FB.

## 2 PATIENTS AND METHODS

### 2.1 Study population

This study was conducted after approval of the local Institutional Review Board. Eligible patients with Prostate Imaging Reporting & Data System 3 (PI‐RADS 3) or greater ROIs were enrolled. Patients with a prior MRI–ultrasound (US) FB or in‐bore MRI‐guided biopsy were excluded. Patients with PSA > 100 ng/ml or radiographic evidence of metastatic cancer, and those taking any type of androgen suppression were excluded. Patients who underwent any prior attempt at definitive local PCa therapy, radiation or otherwise, were also excluded. Patients were excluded from the analysis if they had already been diagnosed with PCa (i.e., undergoing confirmatory biopsy and/or on active surveillance). Agreement and multivariable analyses were performed on a per ROI basis.

### 2.2 Diagnostic imaging

The majority of mpMRIs were performed at 3‐Tesla with an endorectal coil using the technical standards set forth by PI‐RADS v2. All mpMRI, including the few not performed at our institution, were interpreted by two diagnostic radiologists (BF, FC) with extensive mpMRI experience using PI‐RADS v2 criteria. Prostate and ROI contouring was performed using Dynacad software (Invivo, Philips, Best, Netherlands).

### 2.3 Biopsy procedure details

All biopsies were performed by three fellowship‐trained urologic oncologists (JJL, SI, CLA) with extensive experience in fusion and standard TRUS biopsy. One of the surgeons performed FB prior to this study in training, while the other two surgeons had been performing FB for 2 years prior to initiation of the study. Informed consent was obtained, and biopsies were conducted per protocol, not as standard of care. The protocol is as follows. First, a TRUS was performed (BK Medical, Peabody, MA), and two cores from the targeted ROI on MRI were obtained using the CB technique, sampling either a TRUS‐visible hypoechoic ROI corresponding to the region identified by MRI or the general area specified by MRI if no hypoechoic ROI could be identified. Second, the MRI target ROI and prostate contour were co‐registered with the TRUS image using the UroNav FB device (InVivo, Philips, Best, Netherlands), and two cores were obtained from the ROI. If multiple ROIs were present, CB of all ROIs was performed first. FB followed in the standard fashion. Finally, a 12‐core standard sextant systematic biopsy was obtained.

### 2.4 Outcomes

Overall, cancer detection was the primary outcome and defined as any Gleason 3 + 3 or higher found in one or more cores for the ROI on biopsy final pathology. High‐grade cancer detection was a secondary outcome and defined as any Gleason 3 + 4 or higher found in one or more cores for the ROI on biopsy final pathology.

### 2.5 Statistical analysis

McNemar's test was used to analyse overall agreement of any PCa detection between CB and SB.

Agreement was then assessed across specific pre‐biopsy clinical and MRI spatial parameters. These included clinical variables that are known or suspected to affect cancer detection rate, namely, age, ethnicity, family history of PCa, digital rectal exam (DRE) and previous biopsy. We also included MRI characteristics including PI‐RADS score, prostate volume, ROI maximum dimension, transverse dimension, anteroposterior dimension, craniocaudal dimension, and volume, and ROI location (base, apical and anterior). Correlation between agreement for any cancer detection and each variable was tested using chi‐square test, Fisher's test or Wilcoxon rank sum test where appropriate. A *p* value of <0.05 was considered significant.

Variables that significantly affected agreement were then selected for a separate secondary multivariable analysis to compare cancer detection rate between fusion modalities. A generalized mixed‐effect model was used. To compare CB and FB directly, all ROIs were counted twice within the same model. Individual ROIs had a unique identifier, and this was included as a random effect variable. Fusion modality used (CB or FB) was included as a fixed effect. Interactions between significant variables from the above subgroup analysis and fusion modality were tested. R (version 3.63) and SAS version 9.4 were used.

## 3 RESULTS

### 3.1 Patient and tumour characteristics

Ninety patients and 98 ROIs were included in the study. The median age was 68, and median PSA was 7 ng/ml. Sixty‐four percent of patients were diagnosed with any cancer, and 52% of patients were diagnosed with Gleason grade group 2 or higher. High‐grade cancer detection rate for both targeted biopsy techniques amongst all ROIs was 48%. High‐grade cancer detection rate amongst all ROIs was 40% for FB, and 43% for CB. Tables 1A and 1B summarize patient‐level and ROI‐level characteristics. Fifty‐three percent of all patients had a prior negative biopsy.

**TABLE 1A:** Patient‐level demographics

	*N* = 90 patients^a^
Age, years	68 (64, 74)
PSA, ng/ml	7 (6, 11)
PSA density, ng/ml^b^	0.14 (0.09, 0.23)
Positive family history of PC	21 (23%)
Abnormal DRE	33 (38%)
Prior negative biopsy	48 (53%)
Prostate volume on MRI, ml	52 (35, 78)
Highest PI‐RADS score	
3	12 (13%)
4	39 (43%)
5	39 (43%)
Combined Biopsy Gleason Grade^2^	
No cancer	32 (36%)
3 + 3	11 (12%)
3 + 4	14 (16%)
4 + 3	12 (13%)
4 + 4 or higher	21 (23%)

Abbreviations: CB, MRI cognitive fusion biopsy; DRE, digital rectal exam; FB, MRI‐US fusion biopsy; MRI, magnetic resonance imaging; PC, prostate cancer; PI‐RADS, Prostate Imaging Reporting & Data System; PSA, prostate‐specific antigen; US, ultrasound.

^a^ Median (IQR); *n* (%).

^b^ Systematic biopsy and both fusion modalities.

**TABLE 1B:** ROI‐level characteristics

	*N* = 98 ROIs^a^		
Transverse dimension, cm	1.25 (0.90, 1.70)		
Anteroposterior dimension, cm	1.00 (0.70, 1.20)		
Craniocaudal dimension, cm	1.20 (0.78, 1.70)		
Maximum dimension, cm	1.45 (1.00, 1.92)		
Lesion volume, cc	0.70 (0.30, 1.50)		
PI‐RADS score			
3	14 (14%)		
4	44 (45%)		
5	40 (41%)		
Anterior location	20 (20%)		
Highest Targeted Biopsy Gleason Grade	FB + CB	CB	FB
No cancer	43 (44%)	48 (49%)	52 (53%)
3 + 3	8 (8%)	8 (8%)	7 (7%)
3 + 4	17 (17%)	12 (12%)	17 (17%)
4 + 3	15 (15%)	17 (17%)	13 (13%)
4 + 4 or higher	15 (15%)	13 (13%)	9 (9%)

Abbreviations: CB, MRI cognitive fusion biopsy; FB, MRI‐US fusion biopsy; PI‐RADS, Prostate Imaging Reporting & Data System; ROI, region of interest; US, ultrasound.

^a^ Median (IQR); *n* (%).

### 3.2 Concordance

Overall, concordance between FB and CB was 85.7% for all ROIs. FB detected cancer where CB did not in nine ROIs (9%). CB detected cancer where FB did not in five ROIs (5%) (Table 2A). McNemar's test was insignificant (*p* = 0.285). Cohen's kappa coefficient for agreement for any cancer was 0.71 (‘moderate agreement’).^13^

**TABLE 2A:** Overall agreement between cognitive and MRI‐US fusion

Agreement	Any cancer	High‐grade cancer	
Both FB + CB positive	41 (42%)	34 (35%)	
FB negative + CB positive	5 (5%)	8 (8%)	
FB positive + CB negative	9 (9%)	5 (5%)	
Both FB + CB negative	43 (44%)	51 (52%)	
Cohens kappa	0.71	0.73	
*p* value^a^	0.285	0.579	

Abbreviations: CB, MRI cognitive fusion biopsy; FB, MRI‐US fusion biopsy; MRI, magnetic resonance imaging; US, ultrasound.

^a^ McNemars test.

Concordance for high‐grade cancer was 86.7%. FB detected high‐grade cancer where CB did not in five ROIs (5%). CB detected **high‐grade** cancer where FB did not in eight ROIs (8%) (Table 2A). McNemar's test was insignificant (*p* = 0.579). Cohen's kappa coefficient for agreement for any cancer was 0.73 (‘moderate agreement’).^13^

### 3.3 Agreement across pre‐biopsy variables

Agreement was stratified by age, PSA, family history of PCa, DRE, ROI location of base, apex, or anterior, prostate volume, ROI maximum dimension, transverse dimension, anteroposterior dimension, craniocaudal dimension, and ROI volume (Table 2B). There was no significant difference in agreement across selected variables except anterior location of the ROI. Amongst anterior ROIs, agreement between CB and FB was 70% versus 89.7% for non‐anterior ROIs (*p* = 0.035).

**TABLE 2B:** Agreement between cognitive and MRI‐US fusion across variables

	Agreement (*N* = 84 ROIs^a^)	Disagreement (*N* = 14 ROIs^a^)	*p* value^b^
Age, years	68.9 (8.2)	69.6 (7.5)	0.5185
PSA, ng/ml	9.7 (6.5)	7.2 (3.4)	0.1612
PSA density, ng/ml^b^	0.2 (0.2)	0.1 (0.1)	0.1737
Positive family history of PC			
No/NA	63 (84%)	12 (16%)	0.5134
Yes	20 (93.8%)	2 (6.3%)	
DRE			
Normal	49 (83.1%)	10 (16.9%)	0.4674
Abnormal	31 (88.6%)	4 (11.4%)	
Prior negative biopsy			
No	42 (89.4%)	5 (10.6%)	0.3219
Yes	42 (82.4%)	9 (17.6%)	
Prostate volume on MRI, ml	58.5 (33.6)	56.5 (22)	0.6809
PI‐RADS score			
3	13 (92.9%)	1 (7.1%)	0.6849
4 and 5	71 (84.5%)	13 (15.5%)	
Transverse dimension, cm	1.4 (0.8)	1.2 (0.5)	0.2704
Anteroposterior dimension, cm	1 (0.6)	1 (0.4)	0.7786
Craniocaudal dimension, cm	1.3 (0.7)	1.3 (0.6)	0.8271
Maximum dimension, cm	1.6 (0.8)	1.4 (0.6)	0.4924
ROI volume, cc	1.7 (3.4)	1.1 (1.1)	0.864
Base location			
No	62 (88.6%)	8 (11.4%)	0.2146
Yes	22 (78.6%)	6 (21.4%)	
Anterior location			
No	70 (89.7%)	8 (10.3%)	0.0352*
Yes	14 (70%)	6 (30%)	

Abbreviations: CB, MRI cognitive fusion biopsy; DRE, digital rectal exam; FB, MRI‐US fusion biopsy; MRI, magnetic resonance imaging; PC, prostate cancer; PI‐RADS, Prostate Imaging Reporting & Data System; PSA, prostate‐specific antigen; ROI, region of interest; US, ultrasound.

^a^ Median (IQR); *n* (%).

^b^ Systematic biopsy and both fusion modalities.

^*^ Statistically significant (*p* < 0.05).

### 3.4 Multivariable analysis with interaction variables

As anterior location was the only significant variable from the agreement analysis, this was selected and included in the generalized mixed‐effect model as part of an interaction term between anterior location and fusion modality. Neither the modality main effect nor the interaction terms were significant, suggesting neither modality was superior for the detection of PCa in our cohort. As expected, anterior location was statistically significant for PCa detection (*p* = 0.015). The odds ratio of any cancer detection in anterior lesions compared with non‐anterior lesions were 3.06 (95% confidence interval [CI] 1.3, 7.4) (Table 3).

**TABLE 3:** Multivariable analysis of interaction term with anterior lesion location for PC detection

Variable	OR (95% CI)	*p* value
MRI‐US fusion (vs. cognitive)	0.7 (0.4, 1.3)	0.332
Anterior (vs. non‐anterior)	3.1 (1.3, 7.4)	0.015
MRI‐US fusion (vs. cognitive) in anterior lesions	0.6 (0.2, 1.9)	0.411
MRI‐US fusion (vs. cognitive) in non‐anterior lesions	0.9 (0.7, 1.2)	0.478

Abbreviations: CI, confidence interval; MRI, magnetic resonance imaging; OR, odds ratio; PC, prostate cancer; US, ultrasound.

## 4 DISCUSSION

This study found that in a within‐patient protocol, there was moderate agreement for the detection of any PCa between CB and FB all patients with no prior cancer history. The detection rate for high‐grade PCa was 40% for FB, and 43% for CB, which is consistent with prior studies and meta‐analysis that have shown no difference in cancer detection rate between the two modalities.^11^ However, there is heterogeneity amongst prior prospective studies comparing CB with FB; thus, it remains unclear the optimal fusion modality for targeted biopsy, particularly for those that do not have ready access to the technology for FB. The advantage of our within‐patient design is that confounding variables are perfectly balanced, which allows a direct comparison of the technical performance of the two modalities. Our findings that FB and CB perform similarly for the detection of PCa (including high‐grade PCa) reinforce prior studies. There are profound implications for practice patterns (referrals, streamlining care) and overall system cost‐effectiveness if CB were to be adopted as equivalent to FB.

It is a reasonable expectation that there could be certain factors that could favour FB; however, this study did not identify a specific selection criterion that benefits from FB over CB. Stratified by patient and MRI lesion characteristics, agreement was moderate between the two modalities amongst patients with no prior history of cancer except for anterior ROI location, where agreement was nearly 20% lower. Although anterior location was associated with a higher incidence of PCa detection on multivariable analysis, neither FB nor CB detected cancer at a higher rate for anterior lesions. Thus, it is not possible to conclude that either is superior.

One possibility as to why disagreement is higher amongst anterior lesions specifically is that there is more deformation of tissue to reach these targets due to increased distance from needle entry point. The advent of targeted MRI visible lesions has likely not surmounted the challenge of sampling the anterior zone of the prostate with transrectal biopsy approaches. This is reinforced by the higher rate of PCa detection in anterior lesions on multivariable analysis, as patients may have had anterior cancers that were missed with prior negative standard template biopsy as is known to occur commonly.^14^, ^15^ For this reason, urologists must continue to give special attention to patients with MRI visible anterior tumours to avoid diagnostic sampling error. Further studies are needed to determine if these patients may be better served by alternate biopsy techniques, such as MRI‐guided in‐bore or transperineal biopsy, which are becoming increasingly utilized at tertiary referral centers.^16^, ^17^, ^18^, ^19^ As we have seen in this study, FB does not appear to be superior to CB in this setting, but the high rate of disagreement suggests that negative results of any transrectal FB should be interpreted with caution.

### 4.1 Limitations

Our study sampled two cores for each modality for a total of four cores. Multiple groups have shown that four biopsy cores of MRI visible regions will detect more high‐grade PCa compared with one or two cores.^20^, ^21^ Thus, the overall cancer detection rate for individual targeting strategies in our study could be lower than if four cores were taken. However, because our interest was in directly comparing the technical performance of the fusion modalities, each modality should be at an equal disadvantage. It is possible that the discordance amongst anterior ROIs might decrease with increased sampling. More research is needed in order to compare the core for core performance of transrectal fusion approaches and other fusion approaches such as transperineal fusion or MRI in bore‐guided biopsy. Other limitations include the relatively large size of the lesions included in this study, which may have precluded detection of a difference between the two techniques as larger lesions could be more successfully sampled via cognitive FB. Larger lesion size was likely due to the exclusion of active surveillance patients with smaller lesions, and availability of MRI in bore biopsy in our institution, which may have been preferentially used in the case of very small lesions.

Additionally, the performance of FB may have been impacted by the known learning curve for FB.^22^ The success of FB is determined by accurate contouring and co‐registration of the prostate and ROI outlines onto the TRUS images. Small errors in either of these steps, or deformation of the prostate during mpMRI or FB, could result in errors in co‐registration of the target ROI. However, we do not believe that FB or CB had a performance advantage over the other as operators started performing both modalities around the same time period. Thus, this factor is less likely to have an impact when comparing modalities.

This study was performed at a tertiary academic referral centre. Prostate MRIs were interpreted, and target ROIs were marked by specialized radiologists experienced in analysing prostate mpMRI (BF and FC). All three of the urologists in the study were fellowship trained urologic oncologists experienced in TRUS‐guided biopsy (SI, CLA, JJL). Applicability of these findings may be limited for general urology practices or with mpMRI obtained with different techniques and read by general radiologists, where there is known interobserver variability in interpretation.^23^ However, we believe that with the increased diffusion of prostate MRI into practice and most urologists having reasonable existing experience with TRUS, that use of CB techniques should still be feasible and beneficial to those without access to SB.

## 5 CONCLUSION

Cognitive fusion has a similar cancer detection rate of all and high‐grade PCa as MRI‐US fusion via the transrectal approach in a within‐patient comparison, but with moderate agreement. Agreement did not significantly vary across most clinical and MRI variables except amongst anterior ROI locations. Anterior tumours continue to pose a challenge in PCa diagnosis using a transrectal approach. Utilization of cognitive fusion with standard template TRUS biopsy is a viable strategy for urologists without access to MRI‐US fusion technology. Given the limitations of transrectal biopsy for anterior ROIs, alternative biopsy strategies should be considered when the suspicion for PCa persists following negative targeted transrectal biopsy of anterior ROIs.

## AUTHOR CONTRIBUTION

Study concept and design: Mitch Hayes and Jen‐Jane Liu. Analysis and interpretation of data: Mitch Hayes, Luc Boileau, Jacob Grasseur, Solange Bassale, Nicholas Chakiryan. Drafting of the manuscript: Mitch Hayes and Jen‐Jane Liu. Critical revision of the manuscript for important intellectual content: Mitch Hayes, Matthew Wagner, Bryan Foster, Fergus Coakley, Sudhir Isharwal, Christopher Amling, Jen‐Jane Liu. Obtained funding: Jen‐Jane Liu.
